# Biomimetic Nanotechnology Vol. 2

**DOI:** 10.3390/biomimetics7010016

**Published:** 2022-01-14

**Authors:** Ille C. Gebeshuber

**Affiliations:** Institute of Applied Physics, Vienna University of Technology, Wiedner Hauptstrasse 8-10/134, 1040 Wien, Austria; gebeshuber@iap.tuwien.ac.at; Tel.: +43-1-58801-13483; Fax: +43-1-58801-13499

Biomimetic nanotechnology relates to the most basic aspects of living systems, and the transfer of their properties to human applications. Biological materials, structures, and processes are predominantly based on functionalities at the nanoscale. These nanoscale functionalities are often peppered with added components embedded in beautiful hierarchical layers, moving from the nano- to the micro-, through the meso-, and finally to the macroscale [1]. This is of relevance in materials science, medicine, physics, sensor technologies, smart materials science, and various further fields. Biomimetics of nanoscale features of living systems is highly challenging, interesting, and rewarding. Yet, because of the inherent multifunctionality of most biological functions, sometimes it is complicated to isolate specific features that are interesting for potential novel applications in technology. Here, both smart approaches and a focus on properly identifying the underlying principles of Nature are necessary for us to be able to transfer lessons from living systems to technology, science, engineering, and the arts.

The Special Issue “Biomimetic Nanotechnology Vol. 2” [2] of the MDPI Open Access Journal Biomimetics succeeds the Biomimetics Special Issue “Biomimetic Nanotechnology” [3]. The current issue was edited because various important developments have taken place since its predecessor was published, especially in medical biomimetic nanotechnology. Moreover, biomimetics as a field is growing, and has become more consolidated. This issue comprises four original research articles and five review articles. The contributions come from researchers and thinkers in all realms of biomimetic nanotechnology, and features theoretical, experimental, and review contributions from biologists, biomimeticians, chemists, clinicians, engineers, experts in interface and colloid science, food and nutrition specialists, manufacturing specialists, material scientists, microelectronics experts, nanotechnologists, physicists and systems scientists, who are engaged and interested in this fast-growing field.

The specialist fields covered are deep and wide, and comprise original and review articles on biomimetic nanotechnology from dementia research to dentistry (two contributions regard the oral cavity: one focuses on acidic challenges to restorative materials and one on the prevention of detrimental biofilm formation), energy (the generation of hydrogen by water-splitting), nanomembranes (with their vast number of applications), therapies against cancer and multiresistant bacteria, tissue engineering (two contributions: one focuses on the kidney, the other on vascularization) and vaccine design.

The articles highlight the importance of hierarchical materials, nanomaterials, nanoparticles, nanosystems and nanostructures, nanoscale functionalities, programmable materials, safe nanotechnology and tunable materials with nanoscale functionalities in biomimetic nanotechnology.

Especially in the time of the COVID-19 pandemic, there is high interest in vaccines and vaccine design. The review article “Cationic nanostructures for vaccines design”, by the Brazilian author team Carmona-Ribeiro and Pérez-Betancourt [4], approaches this important field from the angle of structuring antigen/adjuvant combinations into four categories. Antigens from the pathogen guarantee an immune response, and adjuvants help create a stronger immune response. The intranasal route is an advantageous mucosal route that allows rapid administration for large populations in the case of pandemics; the development of effective intranasal vaccines is of great interest due to their potential to induce both mucosal and systemic immunity. Cationic nanostructures can further protect the carried antigen for vaccine administration by the oral route, and increase the permanence time of the antigen/carrier assembly at the mucosae, enhancing systemic immunity. The mRNA technology for vaccines has been recognized as a transformative technology to control infectious diseases and to fight cancer. Lipid nanoparticles are a vital component of mRNA vaccines; they protect and transport the mRNA to the place it is needed, and they are currently used in the Pfizer/BioNTech and Moderna COVID-19 vaccines. The huge variety of cationic nanostructured materials available from nanomaterials science may trigger further valuable research on vaccine design with novel cationic nanostructures.

Nanomembranes are the principal building blocks of, essentially, all living organisms. Their artificial counterparts are essential in fields such as separation science, chemical engineering, toxicology, forensics, sensing technology, the life sciences, medicine, the food and drinks industry, process engineering, and green and renewable energy. The Serbian author team Zoran and Olga Jakšić [5] define synthetic nanomembranes as structures with a thickness below 100 nanometers, and a large thickness-to-lateral-size aspect ratio, resulting in, at times, surprising and counter-intuitive phenomena such as extreme toughness combined with extraordinary foldability and stretchability. Their review article “Biomimetic nanomembranes: An overview” critically deals with purely synthetic biomimetic nanomembranes, their fabrication and functionalization. Nanomembranes define boundaries and shapes, and yet can enable complex exchange when properly functionalized. The classes and types of synthetic nanomembranes comprise inorganic nanomembranes, hybrid organic/inorganic nanomembranes, organic nanomembranes and synthetic biological nanomembranes. They can be fabricated top-down, bottom-up, and via exfoliation approaches. Functionalization in biological nanomembranes is mainly via built-in protein structures; in synthetic nanomembranes the toolbox is vastly larger, allowing for the incorporation of various non-biological properties such as plasmonic, magnetic, electrical and optical ones. An example for the functionalization of synthetic biomimetics nanomembranes are water channels inspired by aquaporins that ensure the rapid passing of water combined with the outstanding rejection of undesired ions. The outlook further presents visionary approaches such as biomimetic metamaterials and quantum functionalities.

Decentralized systems, such as those found in social insects and more than 70 different species of bacteria, can coordinate joint behavior and perform complex tasks by using a process called quorum sensing. In this decision-making process, the decentralized systems correlate stimulus and response to population density. Inhibiting the quorum sensing of certain harmful bacteria in the oral cavity by certain molecules can prevent the formation of detrimental biofilm, providing a treatment for dental plaque. Such antibacterial molecules can be encapsulated in nanoparticles, which are then transported via targeted delivery to the teeth and mucins. The structure-giving component of the mucus of organisms such as mucosal surfaces in the oral cavity are also present in saliva. This can provide the patient’s immune system with essential time to fight an infection, and thereby represents an alternative system to antibiotics, against which microbial resistance can arise. The nanostructures can additionally be coated with substances such as chitosan, that react electrostatically with bacterial membranes, providing additional antibacterial activity. In their research article “Characterisation of the interaction among oil-in-water nanocapsules and mucin” [6], a multinational author team from the University of Leeds (UK) investigate a potential new treatment against biofilms that consists of oil-in-water nanocapsules that are coated with chitosan, and loaded with a quorum-sensing inhibitor. The group analyses the interaction with mucin molecules (in a concentration that is comparable to the one in saliva) and the stability of the nanocapsules via dynamic light scattering and asymmetrical flow field-flow fractionation. Such studies form an important knowledge base for the rational design of drug delivery systems in oral environments and shall—in the future—also include investigations regarding the stability and drug-release potential of nanocapsules in more realistic situations.

Intrinsic (such as vomiting) or extrinsic (such as acidic drinks) acidic factors in the oral cavity contribute to the fading of the hydroxyapatite in teeth, leading to caries and potential tooth loss. The demineralizing action of acids on the teeth may lead to loss of the outer enamel. The second article in the Biomimetics Special Issue of Biomimetic Nanotechnology Vol. 2 that deals with the oral cavity is the research article “Exposure of biomimetic composite materials to acidic challenges: Influence of flexural resistance and elastic modulus” [7] written by an Italian team, dealing with the effect of acidic conditions on biomimetic composite materials that are used for restorative purposes. The group exposed five different, conventionally used, restorative, biomimetic composite materials to three different treatments (either storage in distilled water, or storage in a carbonated soft drink for some time, and then in distilled water for some time, or storage in the carbonated soft drink for the whole period). Afterwards, the flexural strength and the elastic modulus of the composites was measured with a universal testing machine. Teeth are not the only studied item influenced by acids: restorative materials (that should, in principle, guarantee good mechanical properties) were subjected to acid-induced corrosion, resulting in changes in their initial characteristics, such as a reduction in microhardness, flexural strength and elastic modulus. Future studies shall be conducted in vivo (allowing the buffering capacity of saliva to be taken into account), on the acidic conditions that naturally appear in the oral cavity.

Biological substitutes for medical applications are an important aspect of tissue engineering. For the development of artificial organs, the combination of natural blood vessels and artificial tissues is of high relevance. In their research article “Fabrication of nanoporous polylactic acid microtubes by core-sheath electrospinning for capillary vascularization” [8], the authors report on the development of base ingredients for future complex synthetic tissues, that allow for the creation of vascular networks in engineering constructs. They use the core-sheath electrospinning process (via which a fiber is formed by simultaneous flow of core and sheath solutions from separate capillaries) to fabricate nanoporous microtubes that mimic the structure of fenestrated capillary vessels. Fenestrated capillary vessels have small pores and are located mainly in places of the body that require considerable exchange between blood and tissues. The microtubes are electrospun and then post-processed. The surfaces and cross-sections of the microtubes are subsequently characterized with scanning electron microscopy. The outer diameter of the artificial vascular channels reported in the article is between one and eight micrometers—this comes close to the natural situation of small human capillaries, which have a diameter of five to ten micrometers. The average size of the surface nanopores ranges from 100 to 800 nm. Furthermore, attachment of the microtubes to human dermal microvascular endothelial cells is preliminarily tested with fluorescence microscopy, indicating the compatibility and potential use as scaffolds for capillary vessel engineering. Future studies will include measurement of the wall thickness, and various solvent mixture combinations.

The use of hydrogen is a possible alternative to the use of fossil fuels. Inspired by the photosystem-II that splits water into hydrogen and oxygen, the authors of the research article “Biomimetic catalysts based on Au@ZnO-graphene composites for the generation of hydrogen by water splitting” [9] report on their studies regarding high-surface area catalysts contributing to artificial photosynthesis. The photosystem-II is a part in the photosynthetic system in plants, algae and cyanobacteria, which stores the energy of light in the form of a redox potential by generating a proton gradient across the thylakoid membranes, i.e., the membranes of the chloroplasts, in which the photosynthetic light reactions and the associated electron transport, as well as proton transport and ATP formation, take place. Hydrogen is abundant, has a high energy yield, can be easily stored, and has environmental compatibility. ZnO is chemically stable, easy to produce and abundant, and provides—in nanosize form—a large surface area, as does the graphene that is used as a co-catalyst, and the gold nanoparticles with diameters of less than 10 nanometers. Their smart combination results in the favored transfer of photogenerated electrons and improved photocatalytic efficiency. The catalysts are characterized with various methods (such as transmission electron microscopy, photoelectron spectroscopy, Raman and X-ray diffraction and UV-vis spectroscopy), and the photocatalytic activity regarding hydrogen production is measured by gas chromatography.

The review article “Biomimetic nanocarrier targeting drug(s) to upstream-receptor mechanisms in dementia: Focusing on linking pathogenic cascades” [10] is written by a single author, the CEO and founder of a US-based company dealing with the continued development of nanoemulsion technology for the “actively targeted” therapy of neuroinflammation, neurodegeneration, and Alzheimer’s disease. Apolipoprotein from the blood can absorb to the surface of certain colloidal lipid nanocarriers that are injected into the blood stream. Apolipoprotein mediates the crossing of the blood–brain barrier. Thereby, these artificial biomimetic nanocarrier particles can transport the pharmaceuticals they carry across the blood–brain barrier, potentially providing a preventive and therapeutic strategy against cognitive decline, dementia and Alzheimer’s disease. Inflammation and oxidative stress, as they occur in microvascular dysfunction, can precede cognitive decline. The author reviews papers that state that endothelial modulation and repair is feasible by the pharmacological targeting of certain endocytic receptors. Oxidative stress and inflammation impair the functionality of high-density lipoproteins (HDL, the so-called “good cholesterol”) in Alzheimer’s disease. Moreover, the gut–brain axis is of high relevance: inflammation in the gut may induce—especially when combined with increased permeability of the blood–brain barrier—proinflammatory cytokine concentration in the brain. Inhibiting these inflammatory cascades may attenuate Alzheimer’s disease. HDL might include some proteins that help inhibit these inflammatory cascades, and could also be an early or even proactive treatment with lipid nanoemulsion vehicles loaded with targeted delivery agents and further drug molecules, allowing for a localized drug treatment of brain tissue in vivo.

The kidney is a paired organ of the urinary system that prepares the urine and regulates water balance, as well as electrolyte balance in vertebrates. It is the most sought-after organ for transplantation. Therefore, a plenitude of attempts is undertaken to replace its function. In their review article “Enhancing kidney vasculature in tissue engineering—current trends and approaches: A review” [11] the author pair from a US American university mainly deal with the current primary obstacle in the development of clinically relevant kidney tissue engineering: the precise formation of blood vessels (i.e., vascularization) for the large-scale growth of whole engineered kidneys for transplantation. Another important aspect in their review is a related comparison between top-down and bottom-up approaches, with regard to vascularization. The general goal of tissue engineering is to develop tissues to assist, maintain and enhance tissue function. Tissue engineering is very successful for avascular structures such as skin or cartilage; however, the engineering of larger organs with intricate structures and vascularization, especially small blood vessels with a diameter less than six millimeters, remains a challenge. The four major kidney tissue engineering methodologies reviewed are: whole kidney tissue engineering (top-down approach), kidney organoids (bottom-up approach), vascular corrosion casts and organs-on-a-chip (microphysiological systems). The authors summarize that although kidney tissue engineering is most challenging and much further research is needed, some highly effective attempts are either already established or hold great promise.

The review article “Learning from Nature: Bioinspired Chlorin-based photosensitizers immobilized on Carbon materials for combined photodynamic and photothermal therapy” [12] by authors from Brazil, gives an extensive review of articles published in this field over the last ten years, comprising the mechanisms and applications of photodynamic and photothermal therapy, carbon materials applied in photodynamic and photothermal therapy and a detailed update on chlorine-based photosensitizers immobilized on carbon materials for photodynamic and photothermal therapy. Photosensitizers induce reactions that may destroy cancerous cells via oxidative pathways and inactivate bacteria, with only a low molar concentration of photosensitizer and low light doses. Certain carbon nanomaterials synergistically support this action. This is also attributed to their large surface area. For example, graphene is a one-atom thin layer of carbon that combines a large surface area, extreme mechanical strength, chemical purity and the possibility of easy functionalization for bioapplications combined with various other supreme properties [13]. Chlorin-based photosensitizers are bioinspired by biomolecules such as chlorophylls. Carbon nanomaterials such as graphene, carbon nanotubes and fullerenes are considered as promising anticancer agents; however, their potential cytotoxicity against healthy cells still poses challenges towards their successful clinical application. Such new technological approaches open new pathways for treating cancer and multi-resistant bacteria.

The guest editor thanks all the contributors, reviewers and the staff of MDPI for their valuable work related to this special issue of the journal Biomimetics, and looks forward to great future developments in the exciting field of biomimetic nanotechnology.

Due to the dynamic developments in the field of biomimetic nanotechnology, and the new interesting contributions we receive, we are glad to inform you that we will continue the successful series of Biomimetics Special Issues in the field of biomimetic nanotechnology with a Volume 3 [14]. The focus of this new special will be the contributions of biomimetics and nanotechnology in the age of a global pandemic.
